# Addressing the Clinical Conundrum of Localization in ACTH-independent Cushing Syndrome With Bilateral Adrenal Nodules

**DOI:** 10.1210/jcemcr/luaf192

**Published:** 2025-09-24

**Authors:** Soo Ling Chan, Rhea Chatterjea, Wei Keat Cheah, Han Boon Oh

**Affiliations:** Department of Medicine, Ng Teng Fong General Hospital, Singapore 609606, Singapore; Department of Medicine, Ng Teng Fong General Hospital, Singapore 609606, Singapore; Department of Surgery, Ng Teng Fong General Hospital, Singapore 609606, Singapore; Department of Surgery, Ng Teng Fong General Hospital, Singapore 609606, Singapore

**Keywords:** ACTH-independent Cushing syndrome, adrenal venous sampling, bilateral cortisol-secreting adrenal adenomas, cortisol lateralization ratio (CLR), aldosterone-corrected cortisol ratio, cortisol/aldosterone ratio (CAR)

## Abstract

ACTH-independent Cushing syndrome is commonly caused by a unilateral adrenal adenoma. However, in cases with bilateral adrenal nodules, localization of the functional lesion is challenging. We present the case of a 26-year-old woman with ACTH-independent Cushing syndrome and concurrent bilateral adrenal nodules. Using dexamethasone-suppressed adrenal venous sampling (AVS), an adrenal vein to peripheral vein cortisol ratio >6.5, and an aldosterone-corrected cortisol lateralization ratio >2.3, the autonomous cortisol secretion was localized to the right adrenal adenoma. She underwent a right posterior retroperitoneal laparoscopic adrenalectomy resulting in clinical and biochemical resolution of hypercortisolism. Postoperatively, she required hydrocortisone therapy, which was successfully discontinued after 4 years. The use of dexamethasone-suppressed AVS, in conjunction with aldosterone-corrected cortisol lateralization ratio, may enhance the diagnostic accuracy of AVS.

## Introduction

Endogenous Cushing syndrome (CS) is characterized by chronic cortisol overproduction, leading to a spectrum of symptoms and significant morbidity and mortality. Primary causes include ACTH-producing pituitary tumors (Cushing disease), ectopic ACTH secretion from nonpituitary tumors, and cortisol-secreting adrenal adenomas or carcinomas. Unilateral adrenocortical lesions are typically the primary cause of ACTH-independent CS. However, contralateral adrenal nodules may also coexist. These bilateral lesions may manifest as a functional adenoma on 1 side with a nonfunctional counterpart, primary bilateral macronodular hyperplasia (PBMAH), bilateral primary pigmented nodular adrenocortical disease (PPNAD), or the rare occurrence of bilateral adrenal tumors (benign or malignant).

Evaluation of bilateral adrenal masses with ACTH-independent hypercortisolism lacks established guidelines, underscoring the importance of shared decision-making and respecting the patient's autonomy to achieve the best possible outcome.

## Case Presentation

A 26-year-old woman presented with complaints of rapid weight gain, rounded facial features, and easy bruising over the preceding 6 months. Despite dietary control and regular exercise, she gained 10 kg over 1 year. Her medical history was notable for anxiety, for which she was treated with fluvoxamine (her only medication). Her brother had an acoustic neuroma, which had been successfully treated, and he remained in remission. The patient denied any family history of endocrine diseases.

On physical examination, she was found to have elevated blood pressure (143/103 mm Hg), a regular pulse (90 beats per minute), and a body mass index of 24.5 kg/m^2^, categorizing her as overweight according to Asia-Pacific criteria. She demonstrated clinical signs consistent with CS including facial plethora, central obesity, dorsocervical fat pads, ecchymoses on the extremities, and thigh striae.

## Diagnostic Assessment

Fasting glucose was mildly elevated at 106 mg/dL (SI: 5.9 mmol/L) (reference range, 72.1-198.2 mg/dL [SI: 4-11 mmol/L]) and her hemoglobin A1C was 5.8% (reference range, 4.5%-6.4%). Hormonal evaluation revealed elevated 24-hour urinary free cortisol of 513.4 μg/day (SI: 1417 nmol/day) (reference range, 4.3-176 μg/day [SI: 12-486 nmol/day]), and an 8 Am cortisol level of 21.9 μg/dL (SI: 604 nmol/L) (reference range, 3.6-19.4 μg/dL [SI: 101-536 nmol/L]) (Table 1). Plasma ACTH was undetectable at <5 pg/mL (SI: <1.1 pmol/L) (reference range, 0-46.36 pg/mL [SI: 0-10.2 pmol/L]). Cortisol levels failed to suppress following both a 1-mg overnight dexamethasone suppression test (DST) at 19.3 μg/dL (SI: 534 nmol/L) and a 2-day low-dose DST at 20.3 μg/dL (SI: 560 nmol/L) confirming ACTH-independent CS. Additional tests ruled out pheochromocytoma, primary aldosteronism (PA), and androgen excess (Table 1).

**Table 1.** Laboratory values

Test	Result	Reference range
**Basic metabolic panel**		
Sodium	142 mEq/L	134-146 mEq/L
	(142 mmol/L)	(134-146 mmol/L)
Potassium	3.7 mEq/L	3.5-5.0 mEq/L
	(3.7 mmol/L)	(3.5-5.0 mmol/L)
Bicarbonate	26 mEq/L	22-29 mEq/L
	(26 mmol/L)	(22-29 mmol/L)
Creatinine	0.50 mg/dL	0.55-1.02 mg/dL
	(44 μmol/L)	(49-90 μmol/L)
Urea	6.72 mg/dL	7.84-21.3 mg/dL
	(2.4 mmol/L)	(2.8-7.6 mmol/L)
Calcium	9.2 mg/dL	8.4-10.2 mg/dL
	(2.30 mmol/L)	(2.10-2.55 mmol/L)
**Endocrine function**		
TSH	0.98 mIU/L	0.35-4.94 mIU/L
	(0.98 IU/L)	0.35-4.94 mIU/L
FT4	0.92 ng/dL	0.70-1.48 ng/dL
	(11.9 pmol/L)	(9.0-19.0 pmol/L)
Fasting glucose	106.0 mg/dL	72.1-198.2 mg/dL
	(5.9 mmol/L)	(4.0-11.0 mmol/L)
HbA1c	5.8%	4.5-6.4%
**Adrenal function**		
Plasma cortisol at 8 Am	21.9 μg/dL	3.6-19.4 μg/dL
	(604 nmol/L)	(101-536 nmol/L)
Plasma ACTH	<5.0 pg/mL	0-46.4 pg/mL
	(<1.1 pmol/L)	(0-10.2 pmol/L)
24-hour UFC	513.4 μg/day	4.3-176.0 μg/day
	(1417 nmol/day)	(12-486 nmol/day)
1-mg overnight DST	19.3 μg/dL	<1.8 μg/dL
	(534 nmol/L)	(<50 nmol/L)
Low-dose DST	20.3 μg/dL	<1.8 μg/dL
	(560 nmol/L)	(<50 nmol/L)
Aldosterone	<4 ng/dL	<21 ng/dL
	(<111 pmol/L)	(<583 pmol/L)
Plasma renin activity	3.6 ng/mL/h	0.6-4.3 ng/mL/h
Urine metanephrine	73.7 μg/day	59.4-280.0 μg/day
	(403 nmol/day)	(325-1530 nmol/day)
Urine normetanephrine	118 μg/day	150-490 μg/day
	(693 nmol/day)	(885-2880 nmol/day)
DHEAS	59.26 μg/dL	18.15-407.41 μg/dL
	(1.6 μmol/L)	(0.5-11 μmol/L)

Values in parentheses are International System of Units (SI).

Abbreviations: DHEAS, dehydroepiandrosterone sulfate; DST, dexamethasone suppression test; FT4, free thyroxine; HbA1c, hemoglobin A1C; UFC, urinary free cortisol.

**Figure 1.**
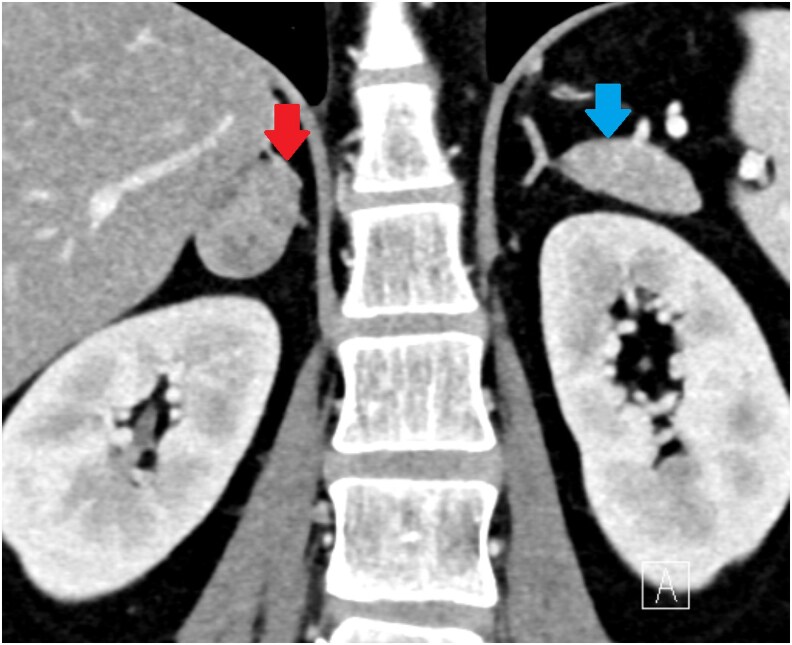
Adrenal enhanced computed tomography (CT) showed a right lipid-poor adrenal nodule (red arrow) with a diameter of 3 cm and a left lipid-poor nodule (blue arrow) with diameters of 1.1 cm (lateral limb). The right nodule was classified as indeterminate based on an absolute washout of 54% and a relative washout of 29%. In contrast, the left adrenal nodule had an unenhanced Hounsfield unit value of 54. This nodule was diagnosed as an adenoma, with an absolute washout of 78% and relative washout of 45%.

Contrast-enhanced adrenal computed tomography (CT) revealed bilateral adrenal nodules: a 3-cm lipid-poor nodule in the lateral limb of the right adrenal gland with unenhanced CT attenuation of 33 Hounsfield units and a 1.1-cm lipid-poor nodule in the lateral limb of the left adrenal gland with unenhanced CT attenuation of 54 Hounsfield units (Fig. 1). Washout analysis characterized the right nodule as indeterminate and the left as an adenoma.

Given the presence of bilateral adrenal nodules, determining the primary source of cortisol excess required careful assessment. Although larger nodules are more often functional, smaller nodules may contribute to hypercortisolism, and nonsecreting incidentalomas may coexist. Adrenal venous sampling (AVS) was performed after an overnight fast on the second day of low-dose dexamethasone administration revealing a right-sided cortisol lateralization ratio (CLR) of 19.5, with an aldosterone-corrected CLR of 10.2. This confirmed a right-sided autonomous cortisol-producing adenoma (Table 2).

**Table 2.** Results of adrenal venous sampling (AVS)

Test	Right AV	Left AV	PV (external iliac vein)
Epinephrine	<25 pg/mL	1914 pg/mL	17 908 pg/mL
	(<136.5 pmol/L)	(10 448.5 pmol/L)	(98 143.8 pmol/L)
Cortisol	340.7 μg/dL	(17.5 μg/dL)	19.2 μg/dL
	(9398 nmol/L)	(483 nmol/L)	(529 nmol/L)
AV/PV cortisol ratio	17.8	0.9	
CLR	19.5		
Aldosterone	8.80 ng/dL	4.60 ng/dL	2.96 ng/dL
	(244 pmol/L)	(128 pmol/L)	(82 pmol/L)
Aldosterone-corrected cortisol ratio	38.5	3.8	
Aldosterone-corrected CLR	10.2		

Values in parentheses are in International System of Units (SI).

Abbreviations: AV, adrenal vein; CLR, cortisol lateralization ratio; PV, peripheral vein.

## Treatment

**Figure 2.**
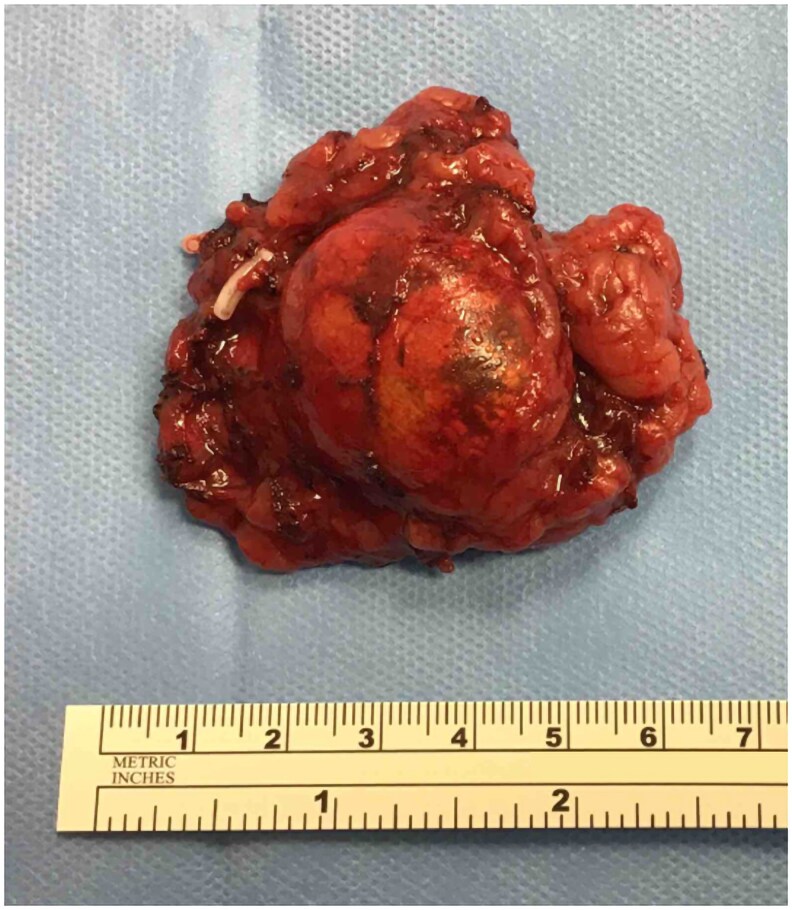
Macroscopic appearance of resected right adrenal adenoma.

The patient underwent posterior retroperitoneal laparoscopic adrenalectomy without complications. Histological analysis confirmed a 2.9-cm benign adrenal adenoma (Fig. 2), with a Weiss criteria score of 0 and a Ki-67 proliferation index of 1%. The patient was prescribed 40 mg daily hydrocortisone replacement therapy on discharge to address adrenal insufficiency.

## Outcome and Follow-up

Following unilateral adrenalectomy, patients commonly experience an initial decline in appetite and quality of life, with symptoms gradually resolving within a year. Although most recover adrenal function within 1 to 1.5 years, this recovery time varies significantly among individuals, ranging from a few months to 4 years, influenced by the severity and duration of preoperative hypercortisolism [1]. Corticotropic axis recovery is defined by a baseline or stimulated cortisol level >18 μg/dL (SI: > 500 nmol/L) [2]. In this case, 8 Am cortisol remained below 3.62 μg/dL (SI: 100 nmol/L) at 1 year postoperatively, confirming persistent adrenal insufficiency. Despite a gradual tapering regime and a short Synacthen test at 2.5 years postoperatively showing a 60-minute cortisol of 17.36 μg/dL (SI: 479 nmol/L), the patient reported persistent postural symptoms following advice to discontinue regular hydrocortisone and only take stress dosing when unwell. She continued to self-titrate her hydrocortisone dose, administering 5 mg daily on weekdays only, while pursuing studies abroad subsequently. Upon her return to Singapore more than a year later, complete recovery was confirmed at 4 years postsurgery with a short Synacthen test peak cortisol of 25.33 μg/dL (SI: 699 nmol/L), leading to the discontinuation of hydrocortisone. The patient's prolonged hydrocortisone dependence may be attributed to several risk factors for delayed recovery, including elevated 1 mg DST >10 μg/dL (SI: ≥276 nmol/L), young age, female gender, and facial plethora at diagnosis [3]. It is noteworthy that the patient's overseas stay may have led to delayed reassessment, overestimating her actual recovery duration.

## Discussion

ACTH-independent hypercortisolism associated with bilateral adrenal nodules presents a diagnostic challenge because of the absence of established guidelines. The differential diagnoses include PBMAH, typically seen in individuals aged 50 to 60 years resulting from aberrant receptor expression; PPNAD, a rare micronodular hyperplasia associated with Carney complex in younger patients (20-30 years); unilateral cortisol-secreting adenoma accompanied by a contralateral nonfunctioning adenoma; and rare cases of bilateral cortisol-secreting adenomas. This discussion focuses on the latter 2 entities because they pose greater challenges in accurately localizing cortisol secretion before adrenalectomy.

In cases of unilateral adrenal masses, lateralization is usually straightforward, with adrenalectomy targeting the affected gland; however, imaging findings may not always correlate with functional assessments. Ueland et al reported that among 39 patients with ACTH-independent CS, 45% of patients with unilateral adrenal nodules on CT exhibit bilateral cortisol hypersecretion on AVS, whereas 29% of patients with bilateral nodules had unilateral secretion [4]. This discrepancy highlights the limitations of CT in identifying the primary source of cortisol excess.

Historically, [6beta-131I] iodomethyl-19-norcholesterol scintigraphy was used for functional localization but presented drawbacks, including lengthy protocols and limited availability [5-8].

18F-fluorodeoxyglucose positron emission tomography-CT (PET-CT) has shown promise in differentiating functional from nonfunctional adrenal masses based on metabolic activity. Patel et al demonstrated that cortisol-secreting lesions exhibit significantly higher standardized uptake value maximum (SUVmax) values (mean 5.9) than nonfunctioning masses (mean 4.2) [9].

Another emerging modality, 68Ga-pentixafor PET-CT, detects CXC chemokine receptor type 4, which is highly expressed by functional adrenal adenomas. Ding et al reported 97.8% sensitivity and 87.5% specificity for lateralizing cortisol overproduction in a study of 64 patients [10]. Using an SUVmax cutoff of 7.1, this test demonstrated a sensitivity of 90.9% and a specificity of 85.3% for distinguishing functional adenomas from nonfunctional lesions [11]. For ACTH-independent CS, an adrenal lesion with an SUVmax > 8.5 yielded 100% sensitivity and 84.9% specificity for diagnosing a cortisol-producing adenoma. Case reports suggest that 68Ga-pentixafor PET-CT may assist in guiding adrenalectomy for PA and achieving Cushing remission [11-13].

Despite these advances, these imaging modalities currently remain investigational and lack widespread availability. Until these modalities become standard practice, AVS remains the gold standard for localizing cortisol hypersecretion in patients with bilateral adrenal nodules. AVS remains crucial in differentiating unilateral from bilateral cortisol hypersecretion; however, its success relies on proper execution and on the availability of reliable reference hormones for accurate cannulation and interpretation.

Young et al pioneered the use of dexamethasone suppression during AVS to suppress endogenous ACTH and ensure that measured cortisol levels reflect autonomous secretion from adrenal cortical cells [14]. Reported suppression regimes include single 1 mg overnight [4], low-dose (0.5 mg every 6 hours), and high-dose (2 mg every 6 hours) dexamethasone. However, the optimal protocol remains unclear.

Cannulation success is confirmed using venography and by evaluating selectivity indices based on cortisol gradients. In PA, an adrenal vein (AV)/peripheral vein (PV) cortisol ratio > 2 (or >5 under cosyntropin stimulation) is commonly used to determine successful cannulation [15]. Epinephrine [16, 17] and metanephrine [18-20] are currently being investigated as alternative markers to define successful adrenal vein cannulation during AVS. Epinephrine thresholds, such as an AV/PV ratio exceeding 27.4, are promising, but may be confounded by stress-induced fluctuations [16]. Metanephrine, a stable metabolite of epinephrine, could offer a more reliable alternative. Dekkers et al found metanephrine selectivity indices were superior to cortisol for confirming cannulation, suggesting an AV/PV threshold of 12 [18], a finding corroborated by Carroll et al [19] as well. Similarly, Kawahara et al reported that a metanephrine selectivity index threshold > 6.45 achieved 100% sensitivity and specificity [20].

In our patient, elevated epinephrine level (1914 pg/mL; SI: 10,447.57 pmol/L) (reference range: <111 pg/mL; [SI: < 605.89 pmol/L]) confirmed successful left cannulation, ruling out misplacement or dilution as potential reasons for low cortisol values or low AV/PV cortisol ratios. However, epinephrine was undetectable on the right, despite a cortisol AV/PV ratio of 17.8, which was consistent with a cortisol-producing adenoma. A common pitfall of right-sided AVS is unintended superselective cannulation, where the catheter is placed too deeply into a small branch of the right adrenal vein instead of the main adrenal vein. This may lead to falsely low or undetectable epinephrine levels, as observed in this patient. This discrepancy underscores the need for more data to validate epinephrine as a reliable marker. Wei et al questioned epinephrine's utility from stress-related fluctuation during catheterization and used aldosterone instead, though their AV/PV ratio cutoffs were not documented [21].

Johnson et al from the Mayo clinic proposed new AVS criteria to differentiate unilateral from bilateral disease in CS with bilateral adrenal nodules [17]. In their series of 25 patients, an AV/PV cortisol ratio of >9 on the affected side and <2.0 on the contralateral side, along with a CLR > 2.3, demonstrated 95% to 100% specificity for unilateral disease. In cases of bilateral disease, an AV/PV cortisol ratio > 5.1 bilaterally and CLR < 1.1 achieved 80% to 90% specificity, refining earlier criteria by Young et al [14]. Establishing reliable criteria is complicated by technical challenges intrinsic to AVS and hormone measurement. Furthermore, Johnson et al found a correlation between right nodule size and cortisol levels, but not on the left [17], potentially because of anatomical differences. The left adrenal vein drains into the renal vein, rendering cannulation more challenging and increasing potential for sample dilution from the phrenic vein, as well as catheter slippage. Sequential sampling in less experienced centers can cause time-based hormone fluctuations. Although aldosterone values can be corrected using cortisol in PA, there is no established standard for cortisol lateralization in CS, highlighting the need for larger studies to validate and refine these criteria for broader clinical application.

We emphasize the importance of correcting cortisol values in AVS for ACTH-independent CS, particularly when the left adrenal contains the culprit nodule. Aldosterone, used in our patient, has limitations because it cannot be fully stimulated with cosyntropin, unlike cortisol in PA. Cosyntropin infusion may also distort results by stimulating normal cortisol production, weakening the reliability of lateralization ratios. Aldosterone levels are also subjected to fluctuations in blood pressure.

Epinephrine has been proposed as an alternative reference hormone. Duparc et al demonstrated its utility in PA cases with concurrent cortisol production, where cortisol correction failed, but epinephrine confirmed lateralization [22]. Using epinephrine may mitigate dilution from the left phrenic vein draining into the left adrenal vein, offering a promising alternative. Table 3 provides a summary of existing literature regarding the diagnostic performance of imaging modalities for cortisol-producing adenomas, and AVS biomarker performance for cannulation and lateralization.

**Table 3.** Summary of the literature on diagnostic performance of imaging modalities for cortisol-producing adenomas and AVS biomarker performance for cannulation and lateralization cited in this case report

Imaging					
Imaging modality	Cutoff threshold (if relevant)	Sensitivity (%)	Specificity (%)	Key comments	References
Adrenal CT		55.0	71.4	45% of unilateral CT nodules showed bilateral secretion; 29% of bilateral CT nodules had unilateral secretion	Ueland et al 2018 [4]
NP-59 scintigraphy		ND	ND	Historical gold standard; Limited by 3-7-day protocols and low spatial resolution	Yu et al 1996; Koga et al 1997; Lumachi et al 2002; Tung et al 2004 [5-8]
18F-fluorodeoxyglucose PET/CT	SUVmax: >5.33	50.0	81.8	Cortisol-secreting masses had a higher mean SUVmax	Patel et al 2016 [9]
68Ga-pentixafor PET/CT	SUVmax: >7.1 SUVmax >8.5	90.9 100.0	85.3 84.9	Detects CXC chemokine receptor type 4 overexpression on functional lesion	Ding et al 2022 [10] Ding et al 2022 [11]
**AVS (cannulation)**					
Epinephrine	AV/PV ratio >27.4 Absolute level > 364 pg/mL (1988 pmol/L)	92.1 92.1	91.3 94.6	Can be confounded by stress-induced fluctuations	Dream et al 2022 [16]
Metanephrine	AV/PV ratio > 12 AV/PV ratio >6.45	98.0 100.0	89.0 100.0	Stable metabolite; unaffected by procedural stress	Dekkers et al 2013; Carroll et al 2023 [18, 19] Kawahara et al 2024 [20]
Aldosterone	AV:IVC ratio Left: 5.58 Right: 6.74	ND	ND	Aldosterone concentrations may remain stable during sampling if PA is excluded	Wei et al 2018 [21 ]
**AVS (lateralization)**					
Cortisol	AV/PV ratio >9 and CLR >2.3 AV/PV ratio >5.1 bilaterally and CLR < 1.1	ND ND	95-100 (For unilateral disease) 80-90 (for bilateral disease)	For use without correction with a reference hormone	Dream et al 2022 [16]
Aldosterone	Aldosterone-corrected CLR>2.3	ND	ND	Adjust for cortisol dilution between adrenal veins	Wei et al 2018 [21]
Epinephrine	Epinephrine-corrected CLR > 4	ND	ND	May be preferred to cortisol, particularly for adenomas expressing CYP11B2, CYP11B1, and CYP17 as both the adenomas and the normal adrenals may produce cortisol	Duparc et al [22]

Abbreviations: AV, adrenal vein; AVS, adrenal venous sampling; CLR, cortisol lateralization ratio; CT, computed tomography; IVC, inferior vena cava; ND, no data; PA, primary aldosteronism; PV, peripheral vein; SUVmax, standardized uptake value maximum.

Finally, genetic evaluation becomes essential when bilateral adrenal nodules are associated with functional syndromes. PBMAH is associated with *ARMC5* mutations, whereas PPNAD is linked to Carney complex and involves *PRKAR1A* mutations. In this patient, genetic testing revealed no pathogenic variants, reducing the likelihood of these conditions but not excluding a genetic predisposition. Further research is needed to explore other genetic contributors.

## Learning Points

- Localization of cortisol secretion in ACTH-independent hypercortisolism in the setting of bilateral adrenal nodules remains challenging, necessitating further refinement of existing diagnostic techniques.
- Although CT and AVS are the current mainstays, emerging imaging modalities such as 68Ga-pentixafor PET-CT hold promise for noninvasive and accurate localization.
- Technical challenges associated with AVS, including cannulation and reference hormone selection, underscore the need for further research aimed at standardizing diagnostic criteria and improving outcomes.

## Contributors

S.L.C. was involved in the diagnosis and management of the patient, manuscript preparation, revision and is accountable for all aspects of work. R.C. was involved in the literature review, manuscript preparation, revision, and is accountable for all aspects of work. H.B.O. was involved in the surgical management of the patient. W.K.C. was involved in the surgical management of the patient. All authors reviewed and approved the final draft.

## Data Availability

Original data generated and analyzed during this study are included in this published article.
